# Loss of the serine protease HTRA1 impairs smooth muscle cells maturation

**DOI:** 10.1038/s41598-019-54807-6

**Published:** 2019-12-03

**Authors:** Ralph Klose, Alexander Prinz, Fabian Tetzlaff, Eva-Maria Weis, Iris Moll, Juan Rodriguez-Vita, Chio Oka, Thomas Korff, Andreas Fischer

**Affiliations:** 1 Division Vascular Signaling and Cancer (A270), German Cancer Research Center, 69120 Heidelberg, Germany; 2 European Center for Angioscience (ECAS), Medical Faculty Mannheim, Heidelberg University, 68167 Mannheim, Germany; 3 Laboratory of Gene Function in Animals, Nara Institute of Science and Technology, 8916-5 Takayama, Ikoma, Nara 630-0192 Japan; 4 Institute of Physiology and Pathophysiology, Heidelberg University, 69120 Heidelberg, Germany; 5 Medical Clinic I, Endocrinology and Clinical Chemistry, Heidelberg University Hospital, 69120 Heidelberg, Germany

**Keywords:** Cardiovascular diseases, Molecular medicine

## Abstract

Vascular smooth muscle cell (VSMC) dysfunction is a hallmark of small vessel disease, a common cause of stroke and dementia. Two of the most frequently mutated genes in familial small vessel disease are *HTRA1* and *NOTCH3*. The protease HTRA1 cleaves the NOTCH3 ligand JAG1 implying a mechanistic link between HTRA1 and Notch signaling. Here we report that HTRA1 is essential for VSMC differentiation into the contractile phenotype. Mechanistically, loss of HTRA1 increased JAG1 protein levels and NOTCH3 signaling activity in VSMC. In addition, the loss of HTRA1 enhanced TGFβ-SMAD2/3 signaling activity. Activation of either NOTCH3 or TGFβ signaling resulted in increased transcription of the *HES* and *HEY* transcriptional repressors and promoted the contractile VSMC phenotype. However, their combined over-activation led to an additive accumulation of HES and HEY proteins, which repressed the expression of contractile VSMC marker genes. As a result, VSMC adopted an immature phenotype with impaired arterial vasoconstriction in Htra1-deficient mice. These data demonstrate an essential role of HTRA1 in vascular maturation and homeostasis by controlling Notch and TGFβ signaling.

## Introduction

Familial small vessel disease is a major cause of stroke and dementia under the age of 60 with *NOTCH3* and *HTRA1* being two of the most frequently mutated genes^1^. In addition, several loss-of-function mutations in *HTRA1* are causative for cerebral autosomal recessive arteriopathy with subcortical infarcts and leukoencephalopathy (CARASIL syndrome)^2^, which is similar to dominantly inherited CADASIL syndrome caused by neomorphic *NOTCH3* mutations^3^. A common feature of these diseases is vascular smooth muscle cell (VSMC) dysfunction on small arterial blood vessels leading to episodes of impaired blood perfusion in certain brain regions.

Since VSMC are critical regulators to maintain vascular homeostasis they show high phenotypic plasticity, where contractile and synthetic VSMC represent the two ends of a spectrum with intermediate phenotypes, which have different morphologies and functions. While naïve VSMC display a synthetic phenotype and are unable to contract but important for maintenance, contractile VSMC control blood flow and pressure. During development, vascular remodeling and injury, synthetic VSMC secrete extracellular matrix proteins and exhibit higher growth rates and migratory activity than contractile VSMC^4^.

Notch signaling is a juxtacrine signaling mode, which controls numerous cell differentiation processes. The signal sending cell expresses Notch ligands of the Delta-like (DLL) and Jagged (JAG) families which activate Notch receptors on adjacent signal receiving cells. The interaction induces receptor cleavage and translocation of the Notch intracellular domain (ICD) to the nucleus, where it interacts with RBP-Jκ and promotes cell type-specific gene expression and induction of the *HES* and *HEY* genes. These encode basic helix-loop-helix (bHLH) transcription factors, which repress gene expression through either binding other bHLH factors or through interacting directly with DNA at promoter regions^5^. In muscle stem cells, HeyL interacts with Hes1 to bind DNA sites with high affinity causing anti-myogenic effects^6^. In VSMC, HES and HEY proteins can inhibit transcription of contractile VSMC marker proteins^7,8^. As such, the effect of Notch signaling on promoting the contractile VSMC phenotype can be counteracted by HES and HEY bHLH factors. This indicates that the outcome of Notch signaling activity is strictly dose-dependent.

Similar to the Notch pathway, TGFβ signaling has also been shown to promote VSCM differentiation^9^. Interestingly, TGFβ signaling can also activate *HEY* and *HES* gene expression in certain cell types^10,11^. Provided that this also occurs in VSMC, HTRA1 might function through controlling expression levels of the *HES* and *HEY* transcriptional repressors via Notch and TGFβ signaling.

Here we aimed at better understanding how the serine protease HTRA1 controls Notch and TGFβ signaling in VSMC and how this affects the VSCM phenotype. HTRA1 is strongly expressed in VSMC and endothelial cells^12,13^ and is known to cleave several intracellular^14–17^ and extracellular substrates^13,18^. Loss of *HTRA1* leads to increased levels of TGFβ1 availability and TGFβ1 signaling, potentially caused by the ability of HTRA1 to cleave either pro-TGFβ1 or GFD6^2,13,19–21^. Recently, we have shown that the Notch ligand JAG1 is a substrate for HTRA1. After cleavage of JAG1 by HTRA1 in the cytosol the remaining JAG1 protein was rapidly degraded^22^. NOTCH3 and JAG1 are both abundantly expressed on VSMC^7,8^. In arterial blood vessels, JAG1/NOTCH3 signaling is required for differentiation, maintenance and contractility of VSMC^23–27^, which is crucial for vasoconstriction and proper organ perfusion. Such blood vessel functions are impaired in familial small vessel disease. Thus, we hypothesized that HTRA1 functions not only to control TGFβ signaling but also to fine-tune NOTCH3 activity in VSMC by regulating the abundance of its ligand JAG1. As both signaling pathways are critically involved in controlling VSMC differentiation^7–9,23,26,28,29^, loss of *HTRA1* may lead to impaired VSMC function and vessel contraction capacity.

## Results

### Loss of *HTRA1* in VSMC increases NOTCH3 signaling

**Figure 1:**
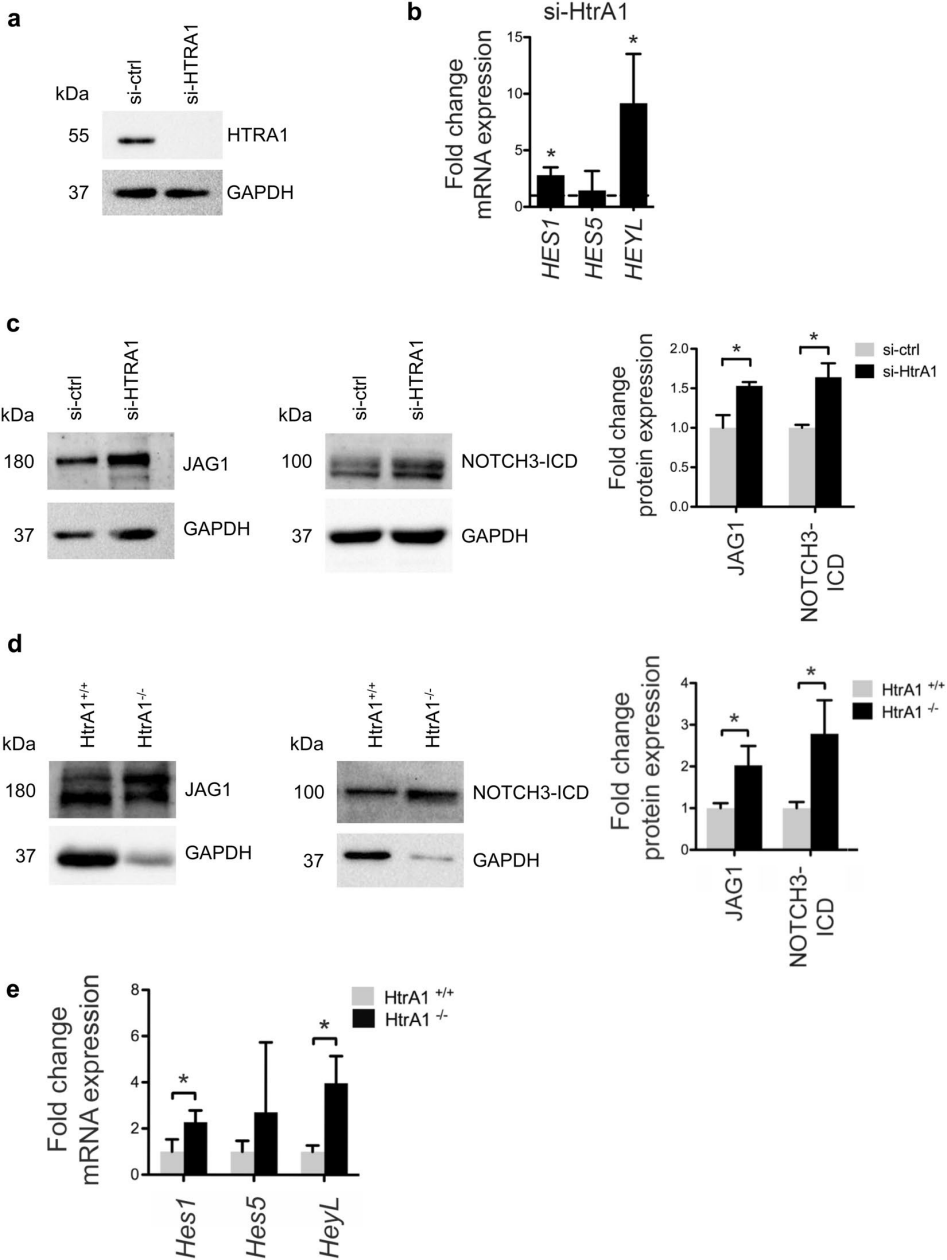
Increased Notch3 signaling activity in *Htra1-*silenced VSMC. (**a**) *HTRA1* was silenced with siRNA. Representative Western blot of HUASMC protein lysates probed with HTRA1 antibody. (**b**) Quantitative real-time PCR analysis of Notch target gene transcripts in HUASMC after silencing *HTRA1* (n = 3). (**c**) Representative Western blot of HUASMC protein lysates probed with anti-JAG1 and anti-NOTCH3-ICD and quantification of band intensities (n = 3). (**d**) Immunoblot of protein lysates derived from mesenteric arteries of *HtrA1*^+/+^ and *HtrA1*^−/−^ mice probed with anti-Notch3-ICD and anti-JAG1 antibody and quantification of band intensities (n = 3). (**e**) Quantitative real-time PCR analysis of Notch target gene transcripts in mesenteric arteries derived from *HtrA1*^+/+^ and *HtrA1*^−/−^ mice (n = 3). Statistical significance was determined by an unpaired student’s t-test (**b**–**e**). *p < 0.05. Bar graphs show mean values, error bars indicate SD.

The similarities between CARASIL and CADASIL syndromes^3^, as well as our recent finding that HTRA1 cleaves the Notch ligand JAG1^22^, prompted us to investigate the potential interplay between HTRA1 and NOTCH3 signaling. Therefore, *HTRA1* was silenced in primary human umbilical artery SMC (HUASMC) using established siRNAs^22^ (Fig. 1a**)**. We observed that silencing *HTRA1* increased mRNA levels of the Notch target genes *HES1* and *HEYL* (Fig. 1b). Higher Notch signaling activity was further evidenced by increased NOTCH3-ICD protein levels and increased JAG1 protein levels (Fig. 1c).

We next isolated arteries from adult *Htra1*^−/−^ mice to verify these data in an *in vivo* model. Compared to wild-type littermate controls, there was an increase in NOTCH3-ICD and JAG1 protein levels in isolated mesenteric resistance arteries from *Htra1*^−/−^ mice (Fig. 1d), indicating augmented NOTCH3 signaling activity. Consistently, we observed a substantial increase in *Hes1* and *HeyL* mRNA levels in mesenteric arteries isolated from *Htra1*^−/−^ mice (Fig. 1e).

### Loss of *HTRA1* in VSMC increases TGFβ signaling

**Figure 2:**
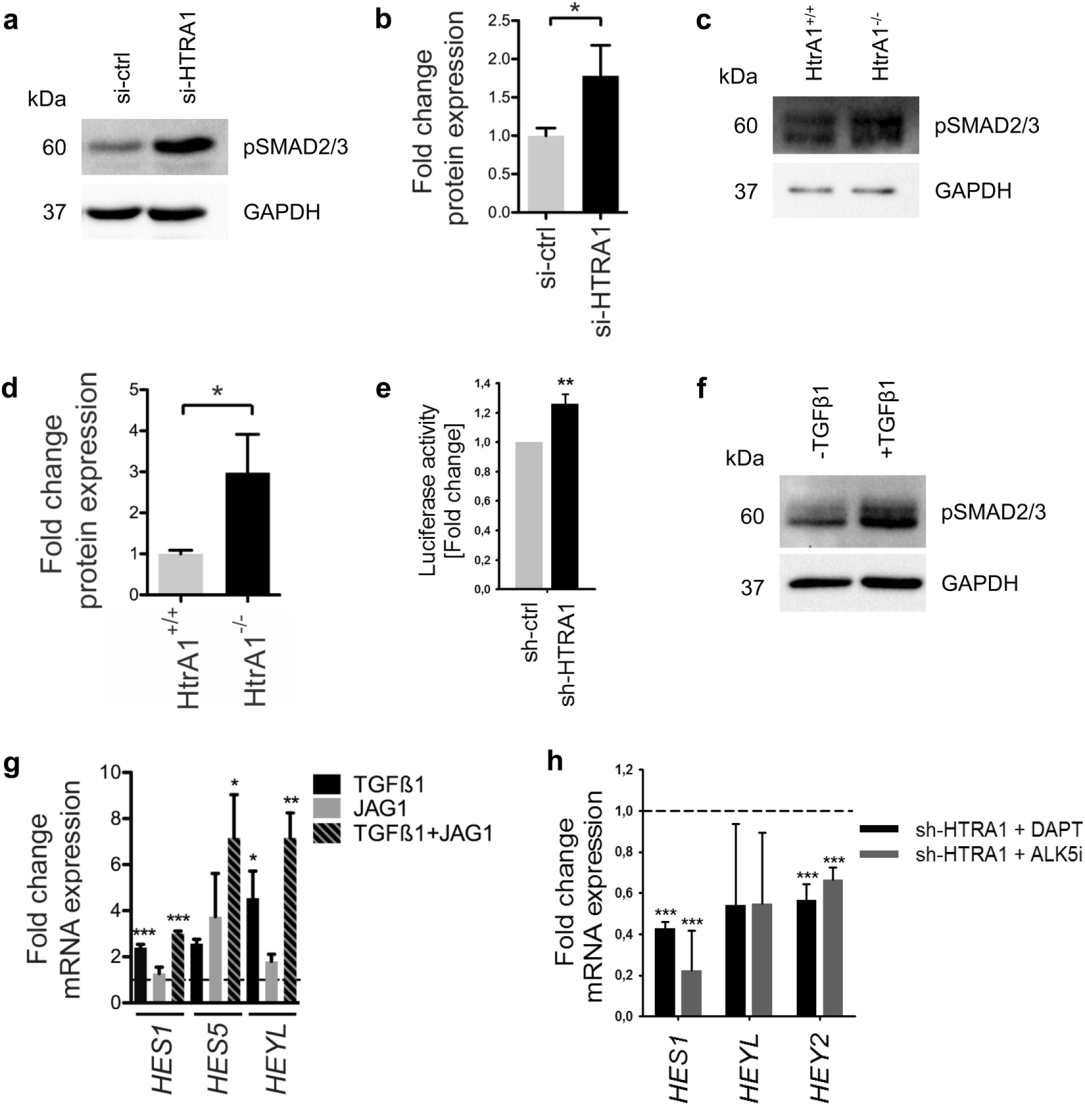
Loss of *HTRA1* in VSMC enforces TGFβ signaling and induces dual induction of *HES* and *HEY* transcriptional repressors synergistically with NOTCH3. (**a**) Immunoblot of phosphorylated SMAD2/3 protein expression after siRNA-mediated silencing of *HTRA*1 in HUASMC. (**b**) Graphical representation of fold change in phosphorylated SMAD2/3 protein level (n = 3). (**c**) Immunoblot of pSMAD2/3 protein level in mesenteric arteries obtained from *HtrA1*^+/+^ and *HtrA1*^−/−^ mice. (**d**) Graphical representation of fold change in pSMAD2/3 protein level (n = 3). (**e**) Co-culture experiment with HUASMC and 3T3 TGFß reporter cells expressing a luciferase cassette with a SMAD responsive promotor (n = 3). (**f**) Western blot of HUASMC protein lysates probed with anti-pSMAD2/3 antibody after stimulation with TGFß1 (5 ng/ml). (**g**) Quantitative real-time PCR analysis of Notch target gene transcripts in HUASMC after TGFß1 stimulation, NOTCH3 stimulation via immobilized JAG1 ligands or combination of TGFß1 and NOTCH3 stimulation (n = 3). (**h**) Quantitative real-time PCR analysis of Notch target gene transcripts in control and *HTRA1*-silenced HUASMC after two hours of TGFß inhibition using 10 µM ALK5 inhibitor (ALK5i, CAS 446859-33-2, Merck) or NOTCH3 inhibition for 16 hours using 25 µM DAPT (Merck). The dashed line denotes gene expression level of control cells (sh-ctrl) treated with DMSO (n = 3). Statistical significance was determined by an unpaired student’s t-test (**b**,**d**,**e**) or by 1-way ANOVA followed by Dunnett post-hoc test (**g**,**h**). *p < 0.05; **p < 0.01; ***p < 0.001. Bar graphs show mean values, error bars indicate SD.

Several reports have shown increased TGFβ1 availability and TGFβ signaling upon loss of *HTRA1*^2,13,19,20^. In HUASMC, silencing of *HTRA1* led to an induction of TGFβ signaling activity as evidenced by elevated levels of phosphorylated SMAD2/3 proteins (Fig. 2a,b). In addition, Smad2/3 phosphorylation levels were higher in isolated arteries from *Htra1*^−/−^ mice when compared to wild-type littermate controls (Fig. 2c,d). Furthermore, when co-cultured with 3T3 reporter cells, silencing of *HTRA1* in HUASMC increased SMAD-dependent luciferase activity (Fig. 2e), indicating that loss of *Htra1* in VSMC results in increased TGFβ signaling in adjacent cells.

### NOTCH3 and TGFβ signaling induces *HES* and *HEY* expression

We and others have shown that TGFβ1 and BMPs can activate *HEY* and *HES* gene expression via SMAD proteins in endothelial cells^10,11^. To examine whether this also applies to VSMC, we treated HUASMC with recombinant TGFβ1 and observed not only increased phosphorylation of SMAD2/3 (Fig. 2f), but also increased transcription of *HES1* and *HEYL* (Fig. 2g). To test this further, we stimulated NOTCH3 signaling in HUASMC using immobilized JAG1 ligands. While stimulation with JAG1 did not significantly affect *HEY* and *HES* gene transcription, combined treatment with JAG1 and TGFβ1 led to an additive induction of *HES1*, *HES5* and *HEYL* expression (Fig. 2g). Of note, the induction of *HES* and *HEY* genes upon silencing of *HTRA1* was fully reverted by inhibiting Notch signaling with the gamma-secretase inhibitor DAPT as well as by inhibiting TGFβ signaling using the ALK5 inhibitor CAS 446859-33-2 (Fig. 2h).

Taken together, these experiments revealed that loss of *HTRA1* leads to over-activation of NOTCH3 and TGFβ signaling, two pathways that synergistically stimulate expression of *HES* and *HEY* transcriptional repressors.

### Loss of *HTRA1* leads to decreased contractile protein expression

Notch signaling is essential for the differentiation of VSMC from precursor cells and for maintenance of the contractile phenotype. JAG1 and NOTCH3 are the most abundantly expressed ligands and receptors respectively on VSMC. Several studies have shown that NOTCH3-ICD activates the transcription of contractile proteins, whereas Notch target genes of *HES* and *HEY* families, encoding transcriptional repressor proteins, inhibit the expression of these contractile VSMC markers^7,8^.

**Figure 3:**
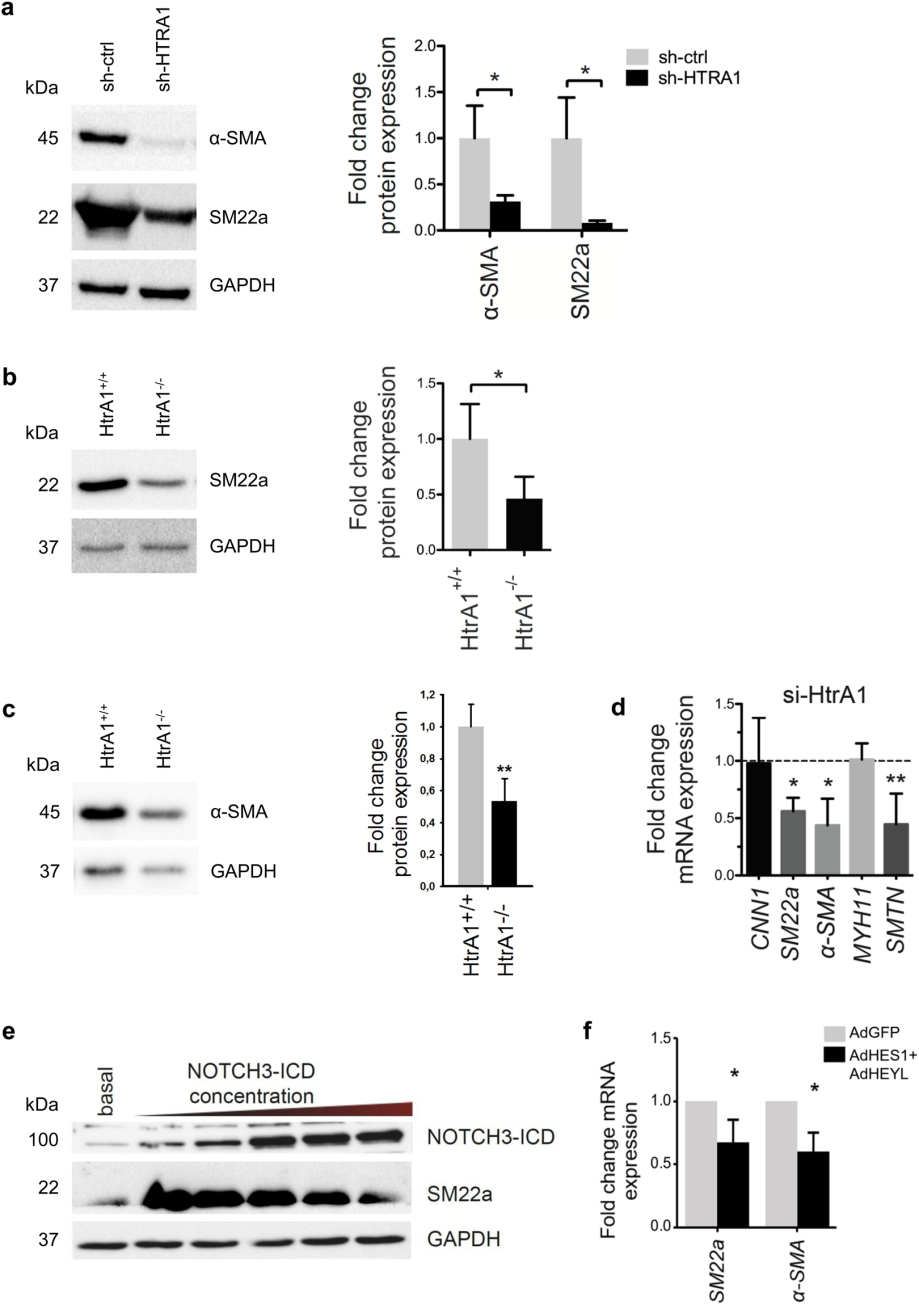
*Htra1* silencing inhibits contractile protein expression. (**a**) Immunoblot and quantification of contractile proteins in control or *HTRA1*-silenced HUASMC (n = 3). (**b**) Immunoblot and graphical representation of fold change in SM22α protein expression in mesenteric arteries (n = 3). (**c**) Immunoblot and graphical representation of fold change in α-SMA protein expression in mesenteric arteries (n = 3). (**d**) Quantitative real-time PCR analysis of contractile gene transcripts in *HTRA1*-silenced HUASMC (n = 3). (**e**) Representative immunoblot of HUASMC protein lysates probed with anti-NOTCH3-ICD and anti-SM22α antibodies. (**f**) Quantitative real-time PCR analysis of contractile gene transcripts in HUASMC overexpressing *HEYL* and *HES1* (n = 3). Statistical significance was determined by an unpaired student’s t-test (**a**,**b**,**c**,**d**,**f**). *p < 0.05; **p < 0.01. Bar graphs show mean values, error bars indicate SD.

Therefore, we analyzed the effect of HTRA1 on expression of contractile proteins. Western blotting revealed a strong reduction of the contractile VSMC phenotype marker proteins α-SMA and SM22α upon silencing of *HTRA1* in HUASMC (Fig. 3a). Similar to cultured VSMC, the protein expression levels of SM22α and α-SMA were diminished in the arterial wall of *Htra1*^−/−^ mice compared to wild-type littermate controls (Fig. 3b,c). Furthermore, the mRNA expression levels of classical contractile VSMC phenotype marker genes *SM22α*, *α-SMA* and *Smoothelin* were diminished after silencing *HTRA1* in HUASCM (Fig. 3d).

To further analyze how hyperactive NOTCH3 signaling contributed to the observed changes seen after *HTRA1* knockdown, we expressed increasing amounts of NOTCH3-ICD in HUASMC. As expected based on previous reports^7,8,23,24,26,28^, moderate over-expression of NOTCH3-ICD led to substantially increased SM22α protein levels (Fig. 3e). This result was however contradictory to what we observed after *HTRA1* silencing (Fig. 3a–d) questioning the relevance of Notch signaling downstream of HTRA1. Interestingly, with higher NOTCH3-ICD expression levels the expression of SM22α decreased again (Fig. 3e), indicating hyper-activation of a negative feedback loop. Indeed, combined over-expression of HES1 and HEYL repressed transcription of *α-SMA* and *SM22α* in HUASMC (Fig. 3f).

Taken together, these data suggest that the strong upregulation of HES and HEY transcriptional repressors by increased Notch and TGFβ signaling upon loss of *HTRA1* leads to repression of genes encoding contractile proteins in VSMC.

### Loss of *HTRA1* in VSMC impairs contractility

**Figure 4:**
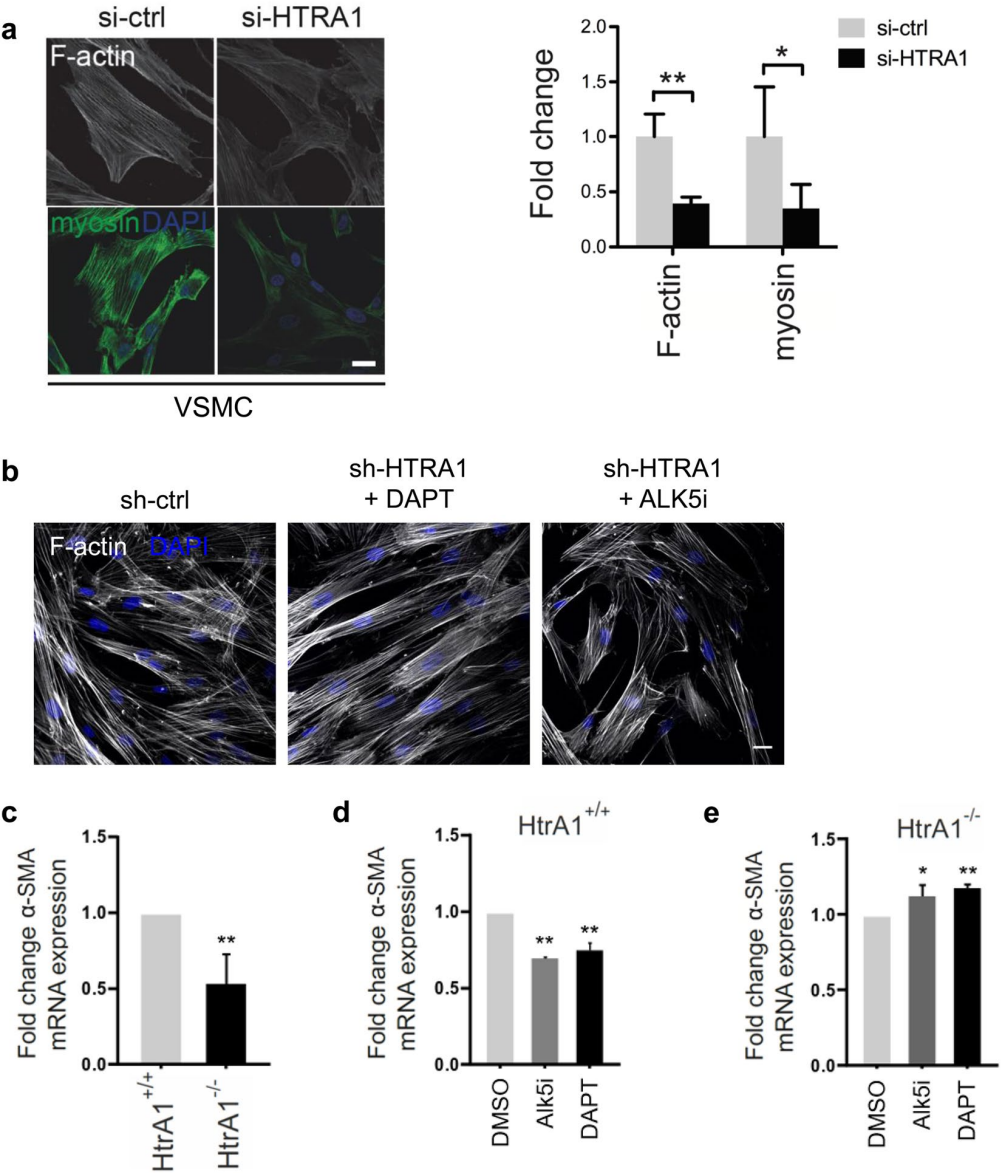
Contractile gene expression in *HTRA1*-deficient VSMC is affected by Notch and TGFß signaling. (**a**) Representative photomicrographs of F-actin and immunolabeled myosin in HUASMC and quantification of F-actin and myosin positive areas (n = 4). (**b**) Representative photomicrographs of F-actin in HUASMC after four hours of TGFß inhibition using 10 µM ALK5 inhibitor (ALK5i, CAS 446859-33-2, Merck) or NOTCH3 inhibition for 16 hours using 25 µM DAPT (Merck) (n = 3). (**c**–**e**) Primary aortic VSMC derived from HtrA1^−/−^ or wildtype littermate controls were cultured until confluence was reached. Quantitative real-time PCR analysis of alpha smooth muscle actin (α-SMA). (**d**,**e**) VSMC were treated with DAPT (25 µM) for 16 hours or Alk5i (10 µM) for two hours before RNA isolation. Treatment with the solvent DMSO was used as control (n = 3). Statistical significance was determined by an unpaired student’s t-test (**a,c,d,e**). *p < 0.05; **p < 0.01. Bar graphs show mean values, error bars indicate SD. Scale bar equals 10 µm.

Impaired vascular contractility contributes to the development of small vessel disease^30^. Therefore, we investigated whether loss of *HTRA1* affects VSMC differentiation and function. Immunofluorescence microscopy revealed substantially less actin and myosin fibers in cultured VSMC after *HTRA1* knockdown (Fig. 4a). The impairment of F-actin formation upon *HTRA1* silencing was restored by inhibition of Notch signaling using DAPT or by inhibition of TGFβ signaling using the ALK5 inhibitor CAS 446859-33-2 (ALK5i) (Fig. 4b).

Next primary aortic VSMC were isolated from HtrA1^−/−^ mice. As expected these had lower *α-SMA* mRNA expression levels compared to aortic VSMC isolated from their wildtype littermate controls (Fig. 4c). While inhibiting Notch activity with DAPT or TGFβ signaling using ALK5i lowered *α-SMA* mRNA expression in wildtype VSMC (Fig. 4d), they partially restored *α-SMA* expression in HtrA1^−/−^ aortic VSMC (Fig. 4e).

**Figure 5:**
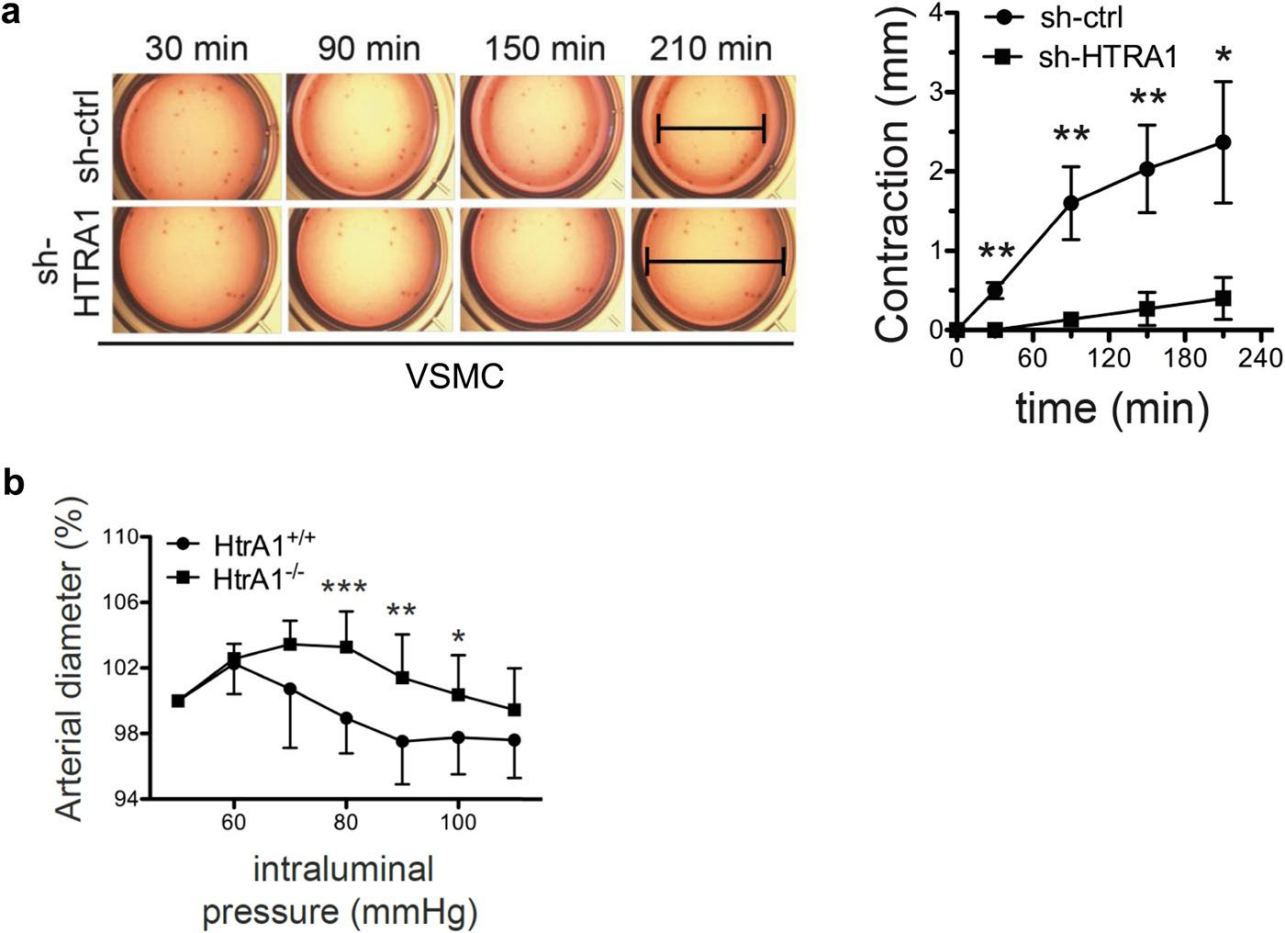
Loss of *HTRA1* in VSMC impairs contractility. (**a**) Cell contraction of control and *HTRA1*-silenced HUASMC at indicated time-points (n = 3). (**b**) Relative increase in diameter of mesenteric artery segments from *HtrA1*^+/+^ and *HtrA1*^−/−^ mice challenged with increasing intraluminal pressure. Diameter of unchallenged arteries was set to 100%. Statistical significance was determined by an unpaired student’s t-test (**a**,**b**). *p < 0.05; **p < 0.01; ***p < 0.001. Bar graphs show mean values, error bars indicate SD.

To further analyze whether *HTRA1* silencing deteriorates cell contractility, we measured the extent of collagen gel contraction by HUASMC. Indeed, cells silenced for *HTRA1* expression were severely impaired in their ability to contract collagen gels when compared to control cells (Fig. 5a).

SM22α is needed to bundle actin filaments that interact with myosin fibers^31^. Although *SM22α*^−/−^ mice are viable, they show impaired vasoconstriction^32,33^. Based on this, we tested how 3^rd^ order branches of mesenteric resistance arteries isolated from *Htra1*^−/−^ mice respond to an increase in blood pressure. Consistent with the *in vitro* data, we observed a less pronounced pressure response in mesenteric arteries from *Htra1*^−/−^ mice compared to control mice, suggesting a switch from contractile to synthetic phenotype (Fig. 5b). We therefore conclude that HTRA1 is required for VSMC maturation and for appropriate contraction of small resistance arteries.

## Discussion

*HTRA1* mutations or altered expression levels of this gene are associated with several diseases, whose pathogenesis is mainly driven by impaired blood vessel function. In humans, *NOTCH3* and *HTRA1* are two of the most frequently mutated genes in familial small vessel disease which is a major cause of stroke and vascular dementia^1^. Moreover, disturbed VSMC function and integrity are main pathogenic factors in small vessel diseases, which impair white matter perfusion leading to dementia^30^. For example, attenuated myogenic responses, reduced caliber of brain arteries and impaired cerebrovascular autoregulation were detected in a mouse model of CADASIL^34^. In autopsies of CARASIL patients, degeneration and loss of VSMC and extracellular matrix proteins, and in addition also severe adventitial fibrosis in small and medium-sized arteries were observed^3^.

Our study shows that the serine protease HTRA1 controls not only TGFβ signaling but also Notch3 signaling, a central regulator of VSMC function. The data suggest that HTRA1 is needed for full VSMC differentiation into the contractile phenotype. In agreement with this, it was very recently reported that VSMC in aged *Htra1*^−/−^ mice adopt the synthetic phenotype and are prone to cell death^35^. Similar to loss of *NOTCH3*^23,26,28^ or overexpression of CADASIL-related *NOTCH3* mutations^36^, loss of *HTRA1* is compatible with normal development of the knockout mouse^37^. However, our data demonstrate that under stressful situations, reduced expression levels of contractile proteins may impair proper blood perfusion.

Furthermore, the data indicate that HTRA1 is required to limit the activity of NOTCH3 and TGFβ signaling in VSMC. TGFβ and NOTCH3 signaling induce VSMC differentiation^7^ and synergistically activate expression of contractile marker proteins^38^ whereupon the Notch transducer RBP-Jκ interacts with Smad2/3 and increases its transcriptional activity. Many promoters of VSMC marker genes contain RBP-Jκ and Smad consensus binding sites in close proximity, suggesting combined action as a transcriptional activator complex^38^. Our data highlight that the outcome of NOTCH3 and TGFβ signaling in VSMC is highly dependent on the signaling strength. While both pathways activate the expression of contractile genes, they also activate *HES* and *HEY* transcriptional repressors. These counteract the Notch-induced up-regulation of contractile proteins like smooth muscle actin^39^. We therefore conclude that the combined over-activation of Notch and TGFβ signaling in *Htra1*-deficient VSMC may counteract the expression of contractile proteins by inappropriately high *HES* and *HEY* gene expression.

In summary, we demonstrated the first functional link between HTRA1 and NOTCH3 signaling. The similar clinical presentation of CARASIL and CADASIL syndromes^3^ and a recent report about aberrant HTRA1 protein levels in a CADASIL mouse model^3^ already suggested such a potential connection. Future work will address how aberrant JAG1 expression influences VSMC biology and how this may be involved in the pathogenesis of small vessel diseases.

## Material and Methods

### Animals and procedures

*Htra1*^−/−^ mice on a C57/Bl6 background were described before^37^. Mice were kept under pathogen-free barrier conditions and animal procedures were performed in accordance with the institutional and national regulation. The local authorities approved all animal experiments.

### Perfusion of isolated mouse arteries

Mice were sacrificed and second/third order mesenteric arteries were isolated and perfused with Tyrode buffer at a longitudinal pressure gradient of 20 mm Hg (70–110 mmHg at the inflow and 50–90 mmHg at the outflow) with a resulting flow of ~0.07 mL/min. Arteries which showed no myogenic response were excluded from the analyses. Pressure-induced changes in vessel diameter were measured using the VediView software (DMT, Copenhagen, Denmark).

### Human vascular smooth muscle cell isolation and cultivation

VSMC were freshly isolated and cultured as previously described^40^. Transfection of cells was done as previously described^22^. Generation of lentivirus and transduction of cells with lentivirus was done as shown elsewhere^41^. To inhibit Notch signaling, cells were incubated with 25 µM DAPT (Merck) overnight. TGFβ signaling was inhibited by adding 10 µM ALK5 inhibitor (CAS 446859-33-2, Merck) for two hours to basal cell medium.

For the co-culture experiment, the same numbers of HUASMC were seeded with 3T3 cells expressing a TGFβ reporter (Luciferase cassette under the control of 12 SMAD binding sites (5′-CAGA-3′) and cultured overnight in basal medium. Luciferase activity was determined using the Luciferase Assay System (E1500, Promega) and a multiwell plate reader (Clariostar, BMG Labtech).

Adenovirus was obtained from abm Inc. (Vancouver, Canada): HES1 (096900 A), HEYL (096954 A). HUASMC were infected with MOI = 50 and cell lysate were prepared 24 hours later.

### Mouse aortic VSMC isolation

Mice were sacrificed by cervical dislocation. After exposing the heart and the descending aorta, fat and adventitia were dissected. The aorta was removed and rinsed in a 60 mm dish with cold PBS containing penicilin and streptomycin and cut into small rings, approximately 1 mm long. Rings were transferred in new 35 mm dishes with 2 ml DMEM with 15% FBS and penicilin/streptomycin containing collagenase at a 10 mg/ml concentration. Rings were incubated overnight. On the next day aortic rings and cells in suspension were transferred to a 15 ml tube, centrifuged (1000 rpm, 5 minutes) and suspended in DMEM with 15% FBS. Cells were incubated until confluence was reached.

### RNA extraction and real-time PCR analysis

Total RNA was isolated using the innuPREP RNA Mini Kit (Analytik Jena). cDNA was synthesized from 1 µg of DNA-free total RNA using M-MLV Reverse Transcriptase (Life Technologies) and random hexamer primers (Life Technologies). Gene-specific transcription levels were determined using the POWER SYBR-Green Mastermix (Life Technologies) and an ABI StepOnePlus real-time cycler (Applied Biosystems). Ct-values were determined with LinReg software and normalization was done with the housekeeping genes OAZ1 (human) and Gapdh (mouse). Primers used were as follows: hOAZ (fw): 5′-gagccgaccatgtcttcatt-3′, hOAZ (rev): 5′-ctcctcctctcccgaagact-3′; mGapdh (fw): 5′-aactttggcattgtggaagg-3′, mGapdh (rev): 5′-acacattgggggtaggaaca-3′; hHES1 (fw): 5′-tcaacacgacaccggataaa-3′, hHES1 (rev): 5′-ccgcgagctatctttcttca-3′; mHes1 (fw): 5′-gtcacctcgttcatgcactc-3′, mHes1 (rev): 5′-tctggaaatgactgtgaagca-3′; hHES5 (fw): 5′-gcatggcccccagca-3′, hHES5 (rev): 5′-acgaaggctttgctgtgctt-3′; mHes5 (fw): 5′-caaggagaaaaaccgactgc-3′, mHes5 (rev): 5′-ggctttgctgtgtttcaggt-3′; hHEYL (fw): 5′-gcaagccaggaagaaacaca-3′, hHEYL (rev): 5′-agaatcctgtcccaccagtg-3′; mHeyL (fw): 5′-tatgatccctctgcgcttct-3′, mHeyL (rev): 5′-catcgatgtgggtcaagaga-3′; hα-SMA (fw): 5′-ctgttccagccatccttcat-3′, hα-SMA (rev): 5′-ccgtgatctccttctgcatt-3′; hSMTN (fw): 5′-cctggtgcacaacttcttcc-3′, hSMTN (rev): 5′-aagacacacttggggtcagg-3′; hCNN1 (fw): 5′-ggagctgagagagtggatcg-3′, hCNN1 (rev): 5′-gtgccaattttgggttgact-3′; hMYH11 (fw): 5′-gtgaacgcactcaagagcaa-3′, hMYH11 (rev): 5′-tattcactggccttggttcc-3′

### Western Blot analysis

Proteins were separated by SDS-polyacrylamide gel electrophoresis and transferred to nitrocellulose membranes (Whatman). The membranes were blocked with 5% skim milk or 3% bovine serum albumin in 0.1% Tween-20 in TBS and incubated with primary antibodies at 4 °C over-night. The membranes were incubated with peroxidase-conjugated secondary antibody for 2 hours at room temperature followed by ECL detection of the antibody. Band intensities were analyzed by the Image lab software (Biorad).

### Antibodies

Anti-JAG1 (Santa Cruz, sc-6011), anti-HTRA1 (R&D, MAB2916), anti-GAPDH (abcam, ab8245), anti-α-SMA (Sigma, C6198), anti-SM22α (abcam, ab137453), anti-NOTCH3 (Santa Cruz, sc-5593), anti-myosin (eBioscience, 14-6400), pSMAD2/3 (Cell Signaling, #8828) were used as primary antibodies. F-actin was stained with Alexa Fluor 568-conjugated phalloidin (Invitrogen).

### Immunofluorescence staining

HUASMC were seeded on glass slides coated with 0.5% gelantine. Cells were washed twice with PBS, fixed with 4% PFA for 10 min, washed three times with PBS, permeabilized with PBS-T (containing 0.1% TritonX) and washed again three times with PBS. After blocking with 3% BSA in PBS, cells were incubated with the primary antibodies over night at 4 °C and secondary antibodies (Thermo Fisher Scientific, 1:400) for 1 hour at room temperature. Sections were counterstained with DAPI, washed three times with PBS and mounted with Fluoromount (S3023, Dako). Confocal images were obtained using an LSM 700 microscope (Carl Zeiss) and analyzed using Fiji software.

### Statistical analysis

Statistical analysis was performed with the Prism 6.0 software (GraphPad Software). Statistical significance was determined by an unpaired student t-test for two samples or by 1-way ANOVA followed by Dunnett post-hoc test when more than 2 groups were compared. Statistical significance is indicated as *p < 0.05, **p < 0.01, ***p < 0.001.

### Study approval

Human umbilical smooth muscle cells were freshly isolated from umbilical cords according to the declaration of Helsinki and with approval of the Heidelberg University ethics review board. Written informed consent from the donor was obtained prior to inclusion in the study. The Animal Care and Use Committee of the Bezirksregierung Baden-Württemberg, Germany, approved all procedures performed on mice.

## Supplementary information

Supplementary figure

## References

[1] Verdura E (2015). Heterozygous HTRA1 mutations are associated with autosomal dominant cerebral small vessel disease. Brain.

[2] Hara K (2009). Association of HTRA1 mutations and familial ischemic cerebral small-vessel disease. N Engl J Med.

[3] Tikka S (2014). CADASIL and CARASIL. Brain Pathol.

[4] Owens GK (1995). Regulation of differentiation of vascular smooth muscle cells. Physiol Rev.

[5] Fischer A, Gessler M (2007). Delta-Notch–and then? Protein interactions and proposed modes of repression by Hes and Hey bHLH factors. Nucleic Acids Res.

[6] Noguchi, Y. T. *et al*. Cell-autonomous and redundant roles of Hey1 and HeyL in muscle stem cells: HeyL requires Hes1 to bind diverse DNA sites. *Development* **146**, 10.1242/dev.163618 (2019). 10.1242/dev.163618 30745427

[7] Boucher J, Gridley T, Liaw L (2012). Molecular pathways of notch signaling in vascular smooth muscle cells. Front Physiol.

[8] Fouillade C, Monet-Lepretre M, Baron-Menguy C, Joutel A (2012). Notch signalling in smooth muscle cells during development and disease. Cardiovasc Res.

[9] Guo X, Chen SY (2012). Transforming growth factor-beta and smooth muscle differentiation. World J Biol Chem.

[10] Itoh F (2004). Synergy and antagonism between Notch and BMP receptor signaling pathways in endothelial cells. EMBO J.

[11] Woltje K, Jabs M, Fischer A (2015). Serum induces transcription of Hey1 and Hey2 genes by Alk1 but not Notch signaling in endothelial cells. PLoS One.

[12] De Luca A (2003). Distribution of the serine protease HtrA1 in normal human tissues. J Histochem Cytochem.

[13] Oka C (2004). HtrA1 serine protease inhibits signaling mediated by Tgfbeta family proteins. Development.

[14] Campioni M (2010). The serine protease HtrA1 specifically interacts and degrades the tuberous sclerosis complex 2 protein. Mol Cancer Res.

[15] Chien J (2009). Serine protease HtrA1 associates with microtubules and inhibits cell migration. Mol Cell Biol.

[16] Grau S (2005). Implications of the serine protease HtrA1 in amyloid precursor protein processing. Proc Natl Acad Sci USA.

[17] Tennstaedt A (2012). Human high temperature requirement serine protease A1 (HTRA1) degrades tau protein aggregates. J Biol Chem.

[18] Tiaden AN, Richards PJ (2013). The emerging roles of HTRA1 in musculoskeletal disease. Am J Pathol.

[19] Friedrich U (2015). Synonymous variants in HTRA1 implicated in AMD susceptibility impair its capacity to regulate TGF-beta signaling. Hum Mol Genet.

[20] Shiga A (2011). Cerebral small-vessel disease protein HTRA1 controls the amount of TGF-beta1 via cleavage of proTGF-beta1. Hum Mol Genet.

[21] Zhang L (2012). High temperature requirement factor A1 (HTRA1) gene regulates angiogenesis through transforming growth factor-beta family member growth differentiation factor 6. J Biol Chem.

[22] Klose, R. *et al*. Inactivation of the serine protease HTRA1 inhibits tumor growth by deregulating angiogenesis. *Oncogene*, 10.1038/s41388-018-0258-4 (2018). 10.1038/s41388-018-0258-4 29713059

[23] Boulos N (2011). Notch3 is essential for regulation of the renal vascular tone. Hypertension.

[24] Domenga V (2004). Notch3 is required for arterial identity and maturation of vascular smooth muscle cells. Genes Dev.

[25] Henshall TL (2015). Notch3 is necessary for blood vessel integrity in the central nervous system. Arterioscler Thromb Vasc Biol.

[26] Li X (2009). Notch3 signaling promotes the development of pulmonary arterial hypertension. Nat Med.

[27] Liu H, Kennard S, Lilly B (2009). NOTCH3 expression is induced in mural cells through an autoregulatory loop that requires endothelial-expressed JAGGED1. Circ Res.

[28] Belin de Chantemele EJ (2008). Notch3 is a major regulator of vascular tone in cerebral and tail resistance arteries. Arterioscler Thromb Vasc Biol.

[29] Low EL, Baker AH, Bradshaw AC (2019). TGFbeta, smooth muscle cells and coronary artery disease: a review. Cell Signal.

[30] Tan RY, Markus HS (2015). Monogenic causes of stroke: now and the future. J Neurol.

[31] Han M (2009). Smooth muscle 22 alpha maintains the differentiated phenotype of vascular smooth muscle cells by inducing filamentous actin bundling. Life Sci.

[32] Je HD, Sohn UD (2007). SM22alpha is required for agonist-induced regulation of contractility: evidence from SM22alpha knockout mice. Mol Cells.

[33] Zhang JC (2001). Analysis of SM22alpha-deficient mice reveals unanticipated insights into smooth muscle cell differentiation and function. Mol Cell Biol.

[34] Joutel A (2010). Cerebrovascular dysfunction and microcirculation rarefaction precede white matter lesions in a mouse genetic model of cerebral ischemic small vessel disease. J Clin Invest.

[35] Ikawati M, Kawaichi M, Oka C (2018). Loss of HtrA1 serine protease induces synthetic modulation of aortic vascular smooth muscle cells. PLoS One.

[36] Dubroca C (2005). Impaired vascular mechanotransduction in a transgenic mouse model of CADASIL arteriopathy. Stroke.

[37] Tsuchiya A (2005). Expression of mouse HtrA1 serine protease in normal bone and cartilage and its upregulation in joint cartilage damaged by experimental arthritis. Bone.

[38] Tang Y (2010). Notch and transforming growth factor-beta (TGFbeta) signaling pathways cooperatively regulate vascular smooth muscle cell differentiation. J Biol Chem.

[39] Tang Y, Urs S, Liaw L (2008). Hairy-related transcription factors inhibit Notch-induced smooth muscle alpha-actin expression by interfering with Notch intracellular domain/CBF-1 complex interaction with the CBF-1-binding site. Circ Res.

[40] Ghosh S (2015). Loss of the mechanotransducer zyxin promotes a synthetic phenotype of vascular smooth muscle cells. J Am Heart Assoc.

[41] Tetzlaff, F. *et al*. MPDZ promotes DLL4-induced Notch signaling during angiogenesis. *Elife* **7**, 10.7554/eLife.32860 (2018). 10.7554/eLife.32860 PMC5933922 29620522

