# Supplementary material for: Loss of the serine protease HTRA1 impairs smooth muscle cells maturation
Source: Sci Rep. 2019 Dec 3;9:18224. doi: 10.1038/s41598-019-54807-6 (PMC6890777; doi:10.1038/s41598-019-54807-6)

Loss of the serine protease HTRA1 impairs smooth muscle cells maturation

Ralph Klose,[1] Alexander Prinz,[1] Fabian Tetzlaff,[1,2] Eva-Maria Weis,[1] Iris Moll,[1] Juan Rodriguez-Vita,[1] Chio Oka,[3] Thomas Korff,[4] Andreas Fischer[1,2,5,#]

[1]Division Vascular Signaling and Cancer (A270), German Cancer Research Center, 69120 Heidelberg, Germany.
[2]European Center for Angioscience, Medical Faculty Mannheim, Heidelberg University, 68167 Mannheim, Germany.
[3]Laboratory of Gene Function in Animals, Nara Institute of Science and Technology, 8916-5 Takayama, Ikoma, Nara 630-0192, Japan.
[4]Institute of Physiology and Pathophysiology, Heidelberg University and Deutsches Zentrum für Herz-Kreislauf-Forschung e.V. (DZHK), Partner site Heidelberg/Mannheim, 69120 Heidelberg, Germany.
[5]Medical Clinic I, Endocrinology and Clinical Chemistry, Heidelberg University Hospital, 69120 Heidelberg, Germany.

[*]**Correspondence**:
Andreas Fischer, M.D.
German Cancer Research Center
A270, Im Neuenheimer Feld 280
69120 Heidelberg, Germany
Email: a.fischer@dkfz.de
Phone: +49 6221 42 4150
FAX: +49 6221 42 4159

Running title: HTRA1 is essential for vascular homeostasis

Supplementary Figure 1

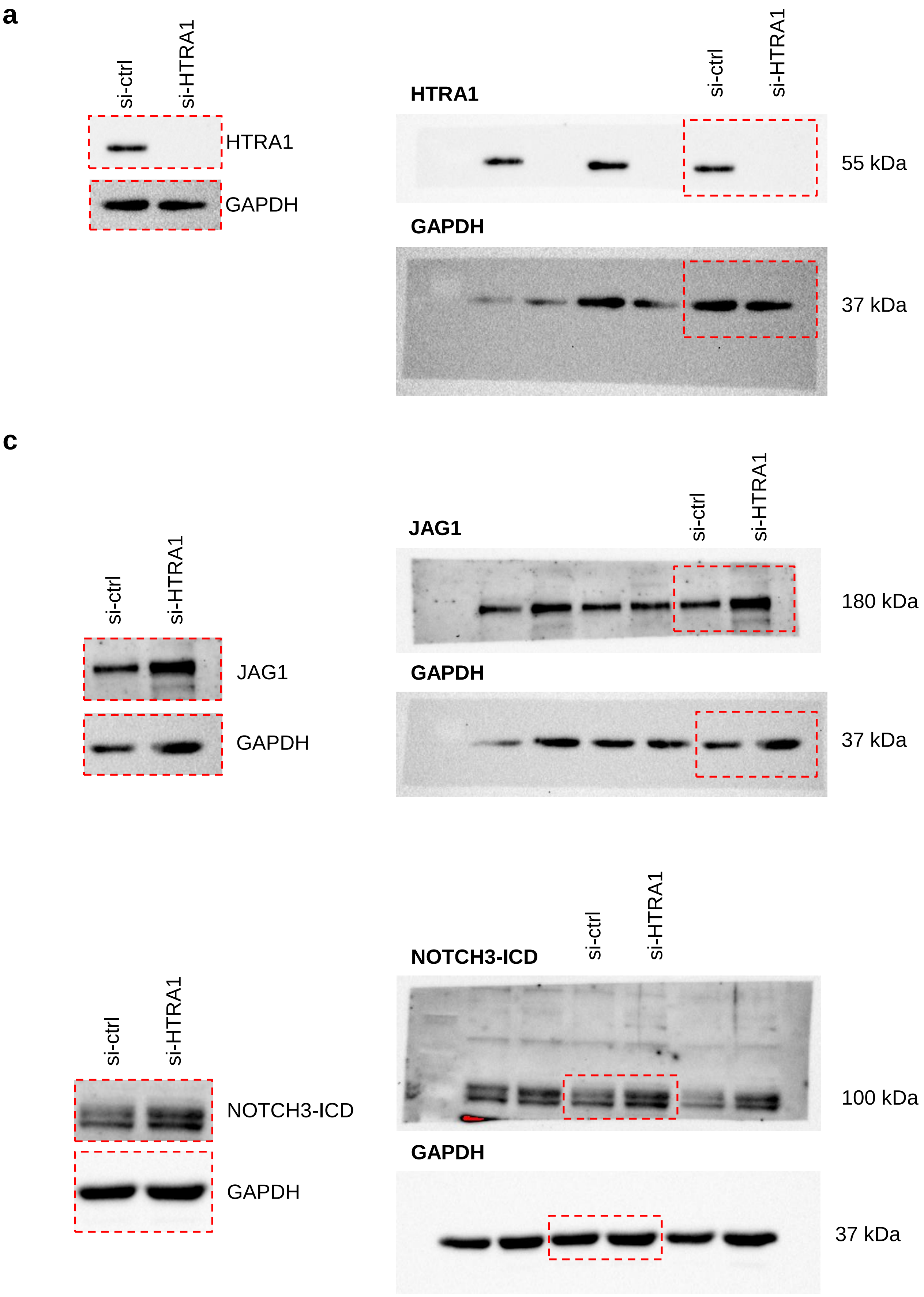

Supplementary Figure 1 (continued)

d

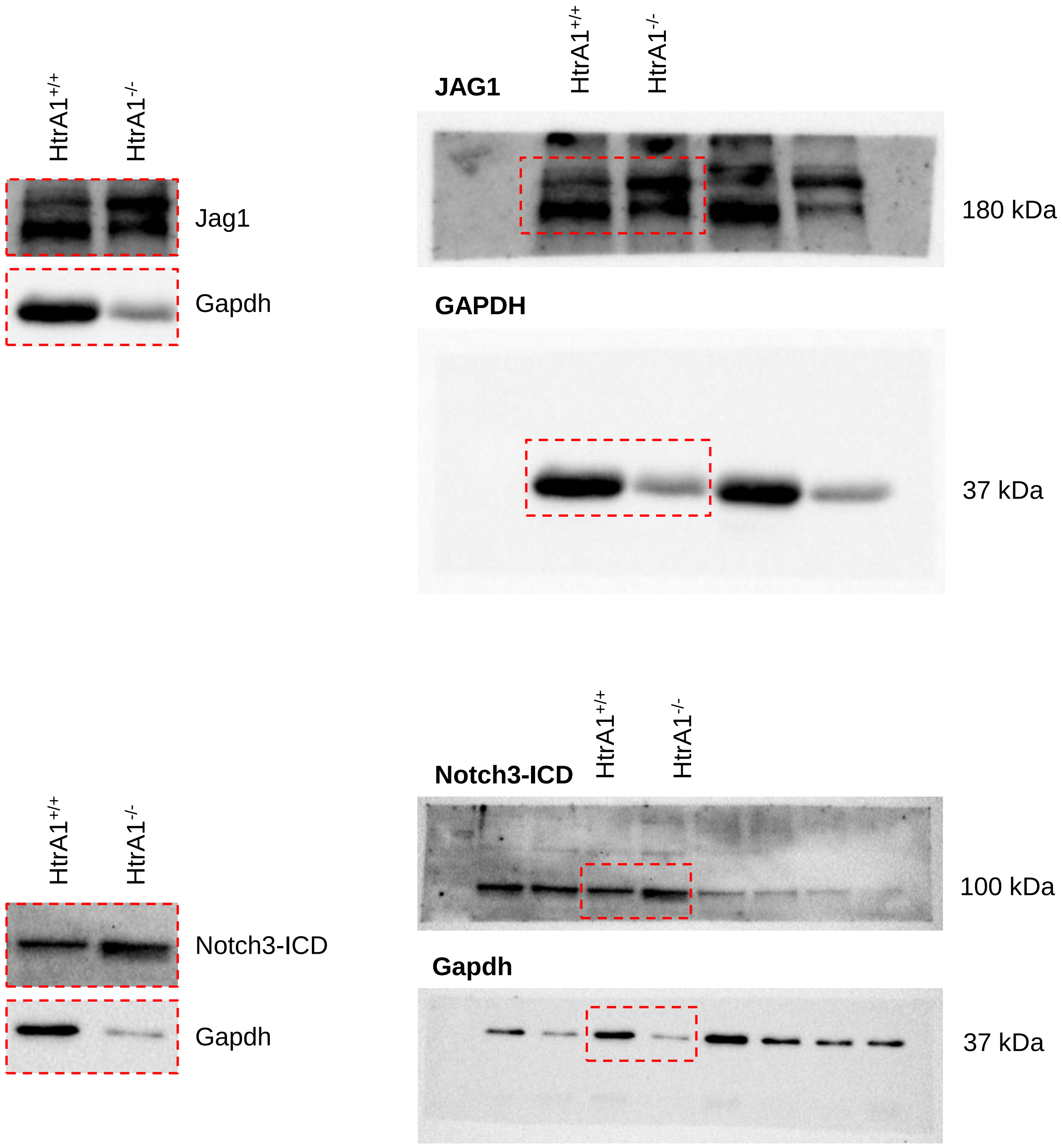

Supplementary Figure 2

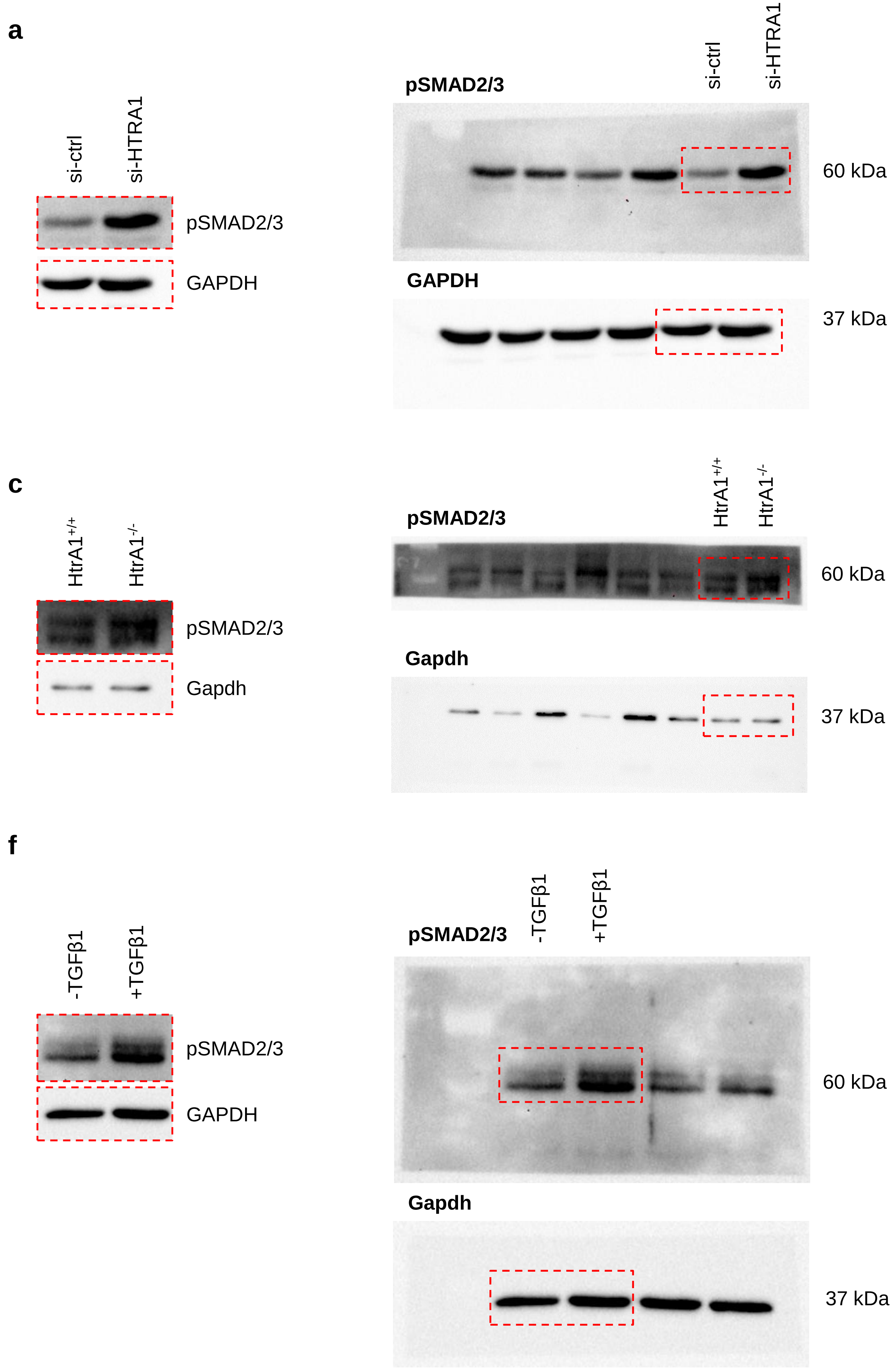

Supplementary Figure 3

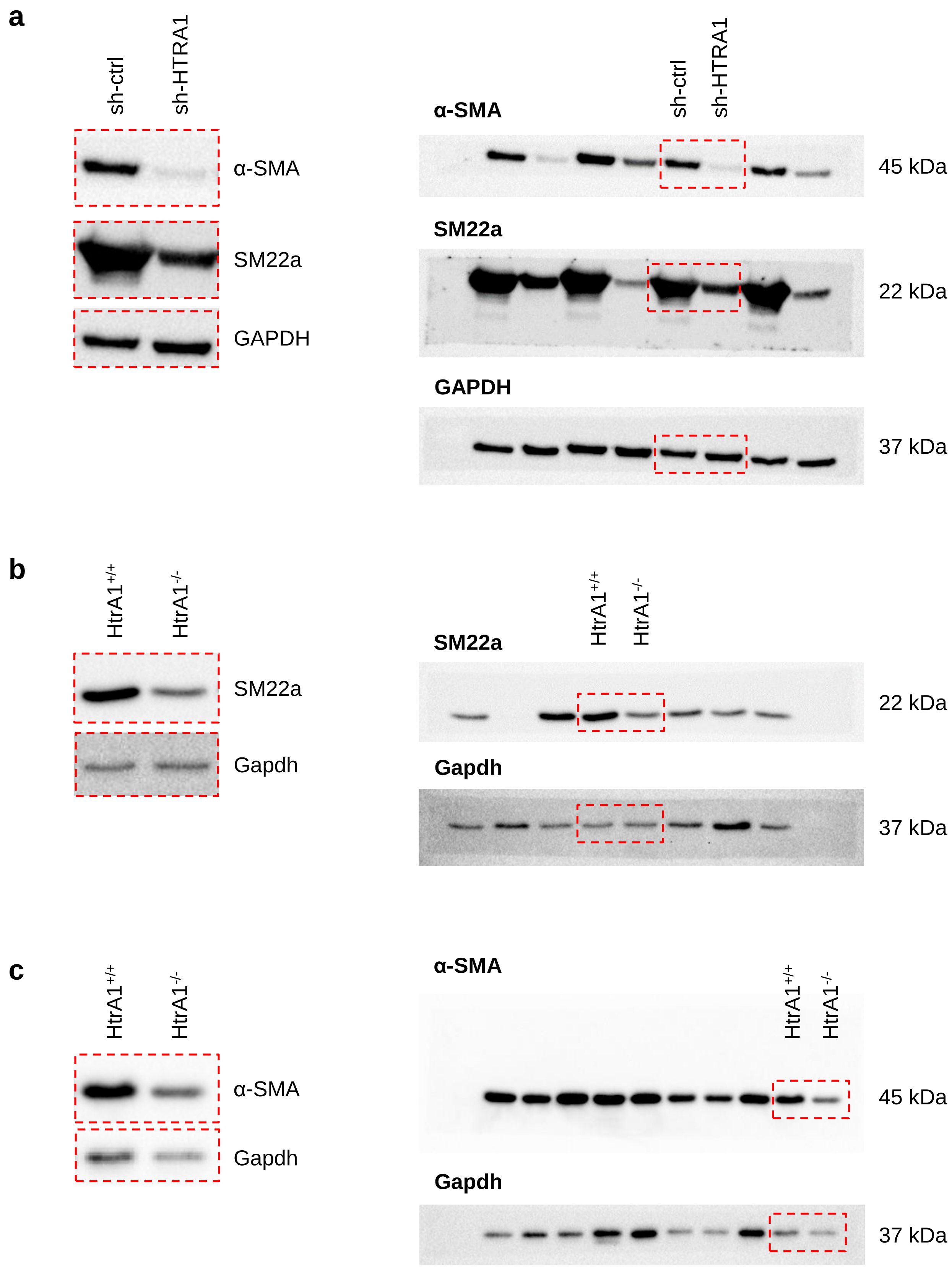

Supplementary Figure 3 (continued)

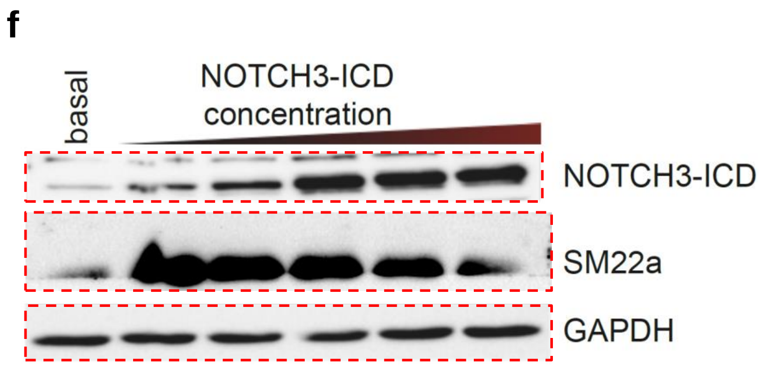

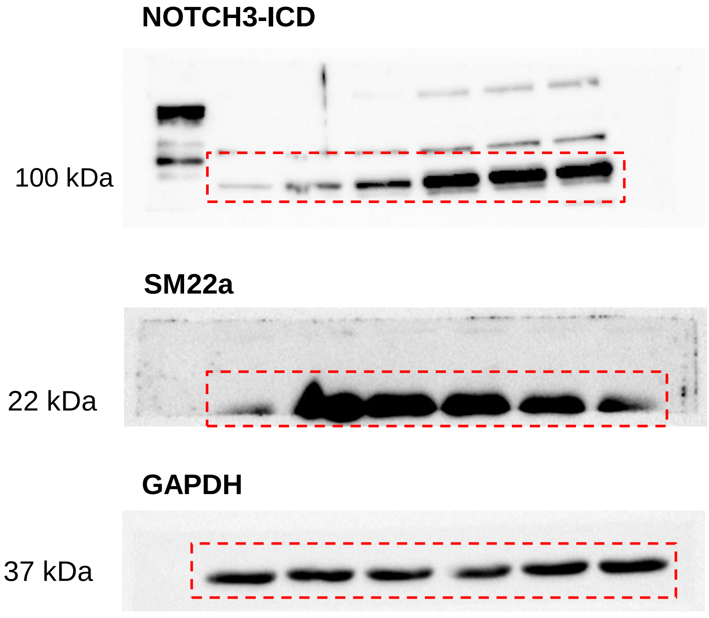

Supplement: Supplementary file 1 — Supplementary figure [file 41598_2019_54807_MOESM1_ESM.pdf]
